# Elevated body mass index and maintenance of cognitive function in late life: exploring underlying neural mechanisms

**DOI:** 10.3389/fnagi.2015.00155

**Published:** 2015-08-18

**Authors:** Chun Liang Hsu, Michelle W. Voss, John R. Best, Todd C. Handy, Kenneth Madden, Niousha Bolandzadeh, Teresa Liu-Ambrose

**Affiliations:** ^1^Aging, Mobility, and Cognitive Neuroscience Lab, Department of Physical Therapy, University of British ColumbiaVancouver, BC, Canada; ^2^Vancouver Health Research InstituteVancouver, BC, Canada; ^3^Djavad Mowafaghian Centre for Brain Health, University of British ColumbiaVancouver, BC, Canada; ^4^Center for Hip Health and Mobility, Vancouver General HospitalVancouver, BC, Canada; ^5^Health, Brain, and Cognition Lab, University of IowaIowa City, IA, USA; ^6^Department of Psychology, University of IowaIowa City, IA, USA; ^7^Department of Psychology, University of British ColumbiaVancouver, BC, Canada; ^8^Department of Medicine, University of British ColumbiaVancouver, BC, Canada

**Keywords:** older adults, default mode network (DMN), mediation analysis, executive functions, body mass index

## Abstract

**Background:** Obesity is associated with vascular risk factors that in turn, may increase dementia risk. However, higher body mass index (BMI) in late life may be neuroprotective. The possible neural mechanisms underlying the benefit of higher BMI on cognition in older adults are largely unknown. Thus, we used functional connectivity magnetic resonance imaging (fcMRI) to examine: (1) the relationship between BMI and functional brain connectivity; and (2) the mediating role of functional brain connectivity in the association between baseline BMI and change in cognitive function over a 12-month period.

**Methods:**We conducted a 12-month, prospective study among 66 community-dwelling older adults, aged 70 to 80 years, who were categorized as: normal weight (BMI from 18.50 to 24.99); overweight (BMI from 25.00 to 29.99); and obese (BMI ≥ 30.00). At baseline, participants performed a finger-tapping task during fMRI scanning. Relevant neural networks were initially identified through independent component analysis (ICA) and subsequently examined through seed-based functional connectivity analysis. At baseline and 12-months, we measured three executive cognitive processes: (1) response inhibition; (2) set shifting; and (3) working memory.

**Results:**Obese individuals showed lower task-related functional connectivity during finger tapping in the default mode network (DMN) compared with their healthy weight counterparts (*p* < 0.01). Lower task-related functional connectivity in the DMN at baseline was independently associated with better working memory performance at 12-months (*p* = 0.02). Finally, DMN functional connectivity during finger tapping significantly mediated the relationship between baseline BMI and working memory at 12-months (indirect effect: −0.155, 95% confidence interval [−0.313, −0.053]).

**Conclusions:**These findings suggest that functional connectivity of the DMN may be an underlying mechanism by which higher BMI confers protective effects to cognition in late life.

## Introduction

Obesity is a complex condition that is characterized by excess body fat and is associated with adverse health outcomes, such as hypertension (Dyer and Elliott, 1989; Kuczmarski et al., 1997), diabetes (Medalie et al., 1974), stroke (Hubert et al., 1983), sleep apnea (Millman et al., 1995), and cancer (Chute et al., 1991; Bostick et al., 1994). Worldwide, there are estimated to be over 1.4 billion overweight (body mass index [BMI] from 25.00 to 29.99 kg/m^2^) and 500 million obese (BMI ≥ 30) adults over the age of 20 years. For the year 2011–2012, age-adjusted estimates suggested among older adults over the age of 60 years in the United States, 71.6% had BMI > 25 and 35.4% had BMI > 30 (Ogden et al., 2014). Thus, the World Health Organization (WHO) considers obesity an epidemic (WHO, 2000).

Current evidence suggests that higher BMI in young and midlife are associated with greater cognitive decline in late life (Elias et al., 2003; Gunstad et al., 2007; Nilsson and Nilsson, 2009; Sabia et al., 2009). The Whitehall II Study, which followed 5131 individuals from young adulthood (25 years old) through midlife (44 years old) to late life (61 years or older), found that higher BMI over young and middle adulthood was associated with lower cognitive performance of executive functions and verbal working memory in late life (Sabia et al., 2009). Moreover, among 1423 adults from age 55 to 88 years, Elias et al. (2003) found that obese individuals showed significantly reduced working memory compared to non-obese individuals over an 18-year observational period. Likewise, Nilsson and colleagues (Nilsson and Nilsson, 2009) followed 2675 middle-age (35–55 years), young-old (60–70), and old-old (75–90) individuals over the span of 10 years and in one cross-sectional analysis they found that in the young-old group, normal weight range individuals performed better in semantic memory compared with overweight individuals (Nilsson and Nilsson, 2009). However, in the old-old group, there was an overall beneficial effect of being overweight for episodic memory (Nilsson and Nilsson, 2009).

This latter finding—along with those from other studies - suggests that elevated BMI in late life may be associated with neuroprotection. These observations, while counterintuitive, do concur with the phenomenon known as the “obesity paradox” (Artham et al., 2008; Childers and Allison, 2010; Flegal et al., 2013), which refers to the observed beneficial association between obesity and health outcomes. For example, a nine-year prospective study by Buchman et al. (2005) with 832 older adults (mean age = 77 years) found that lower baseline BMI was associated with greater cognitive decline. Moreover, subsequent reduction in BMI over the nine-year observation period exacerbated the rate of cognitive decline. Similarly, Van Den Berg et al. (2007) followed 562 older adults over the age of 85 and found that lower BMI was associated with increased rate of decline in both global cognition and executive functions. Thus, although elevated BMI might have neurodegenerative qualities among younger individuals, it appears to have neuroprotective qualities among older individuals. A possible reason for these rather paradoxical observations is that lower BMI and cognitive decline among older adults are both the result of age-related degeneration that affects the body and brain (Grundman et al., 1996; Hu et al., 2002; Boxer et al., 2003; El Fakhri et al., 2003). Unintentional weight loss is included in various criteria for frailty (Bandeen-Roche et al., 2006; Rockwood et al., 2007), and frailty is associated with reduced cognitive performance (Bandeen-Roche et al., 2006).

Despite the growing recognition that higher BMI in older adults may be neuroprotective, few studies to date have assessed the underlying mechanisms. Limited evidence from neuroimaging studies suggests that elevated BMI may moderate the negative effects of Alzheimer's disease (AD) (Grundman et al., 1996; Hu et al., 2002). Specifically, Grundman et al. (1996) found that higher BMI among individuals with AD was associated with greater volumes in the medial temporal cortex, which includes the amygdala, hippocampus, uncus, dentate gyrus, and parahippocampal gyrus, compared with their lean counterparts with AD (Grundman et al., 1996). Another study found that higher BMI among older adults with AD was associated with greater glucose metabolism in the anterior cingulate gyrus and hypothalamus (Hu et al., 2002); this finding in conjunction with studies that report AD-associated reduction in cerebral metabolism (Minoshima et al., 1994, 1997) provide further evidence that higher BMI may have neuroprotective effects in older adults.

The maintenance of cognitive function is supported by multiple neural networks and their mutual connections; both aging and neurodegeneration are characterized by alterations in the coordination of brain networks that support cognitive function (Greicius et al., 2004; Reuter-Lorenz and Lustig, 2005; Persson et al., 2006; Andrews-Hanna et al., 2007; Greicius, 2008; Park and Reuter-Lorenz, 2009; Vidoni et al., 2012). Specifically, among older adults, researchers have identified functional neural networks that are susceptible to aging-related decoupling (Greicius et al., 2004; Reuter-Lorenz and Lustig, 2005; Persson et al., 2006; Andrews-Hanna et al., 2007; Greicius, 2008; Park and Reuter-Lorenz, 2009; Vidoni et al., 2012) and thus, are of interest in the current study. These networks include the default mode network (DMN), the fronto-executive network (FEN), and the fronto-parietal network (FPN). Notably, these three networks have been found to be sensitive to interventions, aging, and BMI status (Mcfadden et al., 2013; Hayes et al., 2014; Gupta et al., 2015; Wijngaarden et al., 2015). Functionally, the DMN demonstrates greater activity during a task-free resting-state and shows less activation during goal-oriented cognitive processes. The DMN is believed to be involved in self-referential thoughts and autobiographical memory retrieval (Andrews-Hanna et al., 2007; Buckner et al., 2008). The FEN is involved in executive function-related cognitive processes and the maintenance of an extended cognitive state during task performance (Dosenbach et al., 2006; Seeley et al., 2007). The FPN is involved in attentional control (Fogassi and Luppino, 2005; Seeley et al., 2007; Sridharan et al., 2008) and may potentially serve as an internal switch that displays synchronous activity with the DMN depending on the task at hand (Spreng et al., 2010; Campbell et al., 2012).

Thus, functional connectivity Magnetic Resonance Imaging (fcMRI) has become an important tool to provide insight into functional coherence of various areas of the brain—in the presence and in the absence of overt behavior or disease. The latter is important within the context of dementia research as it is a disease with a long preclinical phase (Morris, 2005). Moreover, as opposed to changes in brain structures that can only be observed over a longer period of time, functional connectivity allows researchers to examine changes in the brain that occur within minutes of administering the stimulus.

**Figure 1:**
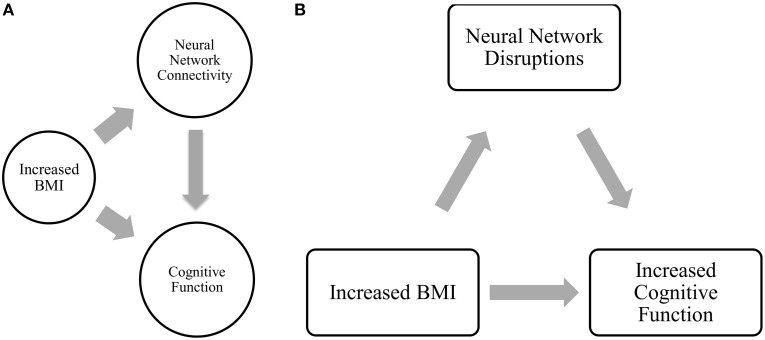
**(A)** Relationship between BMI, network connectivity, and cognitive function. **(B)** Proposed relationship model tested in current analysis.

Moreover, of the few neuroimaging studies that explored the neural mechanisms underlying the association between BMI and cognitive outcomes, the majority were cross-sectional (Grundman et al., 1996; Wang et al., 2001; Hu et al., 2002). Yet, longitudinal data are essential to gaining a greater understanding of how higher BMI may maintain cognitive function among older adults over time. Thus, we conducted a 12-month prospective exploratory study among 66 community-dwelling older adults aged 70 to 80 years, using fcMRI to examine: (1) the relationship between BMI and functional brain connectivity of the DMN, FEN, and FPN (Figure 1A); and (2) the mediating role of functional brain connectivity in the association between baseline BMI and change in cognitive function over the 12-month study period (Figure 1B). We expected to observe differences in functional network connectivity between older individuals with different BMI and that these differences are independently associated with change in cognitive function over time.

## Materials and methods

### Study design and participants

A 12-month prospective exploratory fcMRI study was conducted. We recruited 66 community dwelling older adults from metropolitan Vancouver region via newspaper advertisements. Individuals were eligible if they: (1) were aged 70 to 80 years; (2) scored > 24/30 on the Mini-Mental State Examination (MMSE) (Cockrell and Folstein, 1988); (3) were right hand dominant as measured by the Edinburgh Handedness Inventory (Oldfield, 1971); (4) were living independently in their own homes; (5) had visual acuity of at least 20/40, with or without corrective lenses; and (6) provided informed consent. We excluded those who: (1) had a neurodegenerative disease, stroke, dementia (of any type), or psychiatric condition; (2) were taking psychotropic medication; (3) were living in a nursing home, extended care facility, or assisted-care facility; or (4) did not meet MRI scanning requirements. The study was approved by the Vancouver Coastal Research Health Institute and University of British Columbia's Clinical Research Ethics Board.

### Measurement

All measures, with the exception of neuroimaging as well as comorbidity and depression, were assessed at both baseline and 12-months. All assessors were trained and standardized protocols were used.

#### Anthropometry and categorization based on BMI

Standing height was measured to 0.1 cm, and weight was measured to 0.1 kg. Each participant was categorized as: (1) normal weight (BMI of 18.50–24.99); (2) overweight (BMI of 25.00–29.99); and (3) obese (BMI ≥ 30.00) according to the BMI classification by the WHO (WHO, 1995).

#### Global cognition

Global cognition was assessed using the mini-mental state examination (MMSE) (Cockrell and Folstein, 1988) and the Montreal Cognitive Assessment (MoCA) (Nasreddine et al., 2005). The MMSE and MoCA are 30-point tests that cover multiple cognitive domains. Originally, a score of < 24/30 on the MMSE indicated cognitive impairment (Cockrell and Folstein, 1988). However, recent evidence suggest that a cut-off score of 27 (sensitivity of 0.69; specificity of 0.91) or 28 (sensitivity of 0.78; specificity of 0.78) on the MMSE may be more ideal for identifying individuals with cognitive impairment (O'Bryant et al., 2008). The MoCA have excellent internal consistency and test-retest reliability. It was reported to accurately identify individuals with mild cognitive impairment using a cut-off score of < 26/30 (Nasreddine et al., 2005).

#### Comorbidity and depression

We assessed comorbidities with the Functional Comorbidity Index (FCI) (Groll et al., 2005). The FCI is a questionnaire containing 18 items; and the sum of the 18 items reflects the total number of comorbidities associated with physical functioning (Groll et al., 2005). We used the 15-item Geriatric Depression Scale (GDS) (Yesavage et al., 1982; Yesavage, 1988) to assess individuals' mood; a score ≥ 5 indicates depression (Van Marwijk et al., 1995).

#### Executive functions

For the current study, we focused on three domains of executive functions: selective attention, set-shifting, and verbal working memory. To assess these functions, we administered the Stroop Test (Graf et al., 1995), the Trail Making Tests (Part A and B) (Spreen and Strauss, 1998), and the verbal Digit Span Test (forward and backward; Wechsler, 1981), respectively.

The Stroop Test (Graf et al., 1995) consists of three conditions. The first condition (Stroop 1) requires the participants to verbally read out words printed in black ink. The second condition (Stroop 2) requires the participants to verbally read out the printed color of colored-X's. The final condition (Stroop 3) requires the participants to disregard the meaning of the color-words and verbally name the printed color of color-words that may have been displayed in incongruent ink (e.g., the word “RED” printed in blue ink). The total time participants took to finish each of the three conditions was recorded and difference in time between the third condition and second condition (Part 3 minus Stroop 2) was calculated. Larger time difference reflects poorer selective attention performance.

The Trail Making Tests (Part A and B) (Spreen and Strauss, 1998) consists of two conditions and requires the participants to: (1) sequentially draw lines that connect numbers in order (Part A); or 2) draw lines in alternating sequence connecting numbers and letters (Part B). The total time participants used to complete each condition was recorded and time difference between the two conditions was calculated (Part B minus Part A). Larger time difference reflects poorer set-shifting ability.

The verbal Digit Span Test (Wechsler, 1981) consists of two conditions (forward and backward) and requires the participants to verbally recite a list of random numbers that progressively increase in length in the same order (forward) or reversed order (backward) that was initially read to them. The total number of correct cases for each condition was recorded and a difference score (forward minus backward) was calculated. Larger difference score reflects poorer verbal working memory.

### Functional MRI (fMRI)

The following sections contain details on scanning protocol and parameters as well as analysis pipeline that were described in our previous work (Hsu et al., 2014).

All fMRI was performed at the University of British Columbia (UBC) MRI Research Center located at the UBC Hospital on a 3.0 Tesla Intera Achieva MRI Scanner (Phillips Medical Systems Canada, Markham, Ontario) using an 8-channel SENSE neurovascular coil. The fMRI consisted of 166 dynamic images of 36 slices (3 mm thick) with the following parameters: repetition time (TR) of 2000 ms, echo time (TE) of 30 ms, flip angle (FA) of 90°, field of view (FoV) of 240 mm, acquisition matrix 80 × 80. The high resolution T1 images were acquired using the following parameters: 170 slices (1 mm thick), TR of 7.7 ms, TE of 3.6 ms, FA of 8°, FoV of 256 mm, acquisition matrix of 256 × 200.

#### Motor task

During the fMRI scan, participants performed a simple finger tapping motor test that allows the examination of functional connectivity of networks under different conditions. While simple motor tasks have been shown to be able to elicit strong DMN connectivity patterns (Greicius et al., 2004), it still provides a degree of cognitive challenge that results in task-related deactivation of the DMN relative to complete rest (Hao et al., 2013). In addition, this type of motor task is sensitive to differentiating older adults with AD from those with other types of dementia (Arnold et al., 2005; Grady et al., 2010). Also, evidence suggests that performing continuous task may elicit functional network connectivity different from those generated under task-free conditions in resting-state (Hampson et al., 2004), hence offering additional insight into the behavior of neural networks.

The motor task contains three conditions: finger tapping with left hand, finger tapping with right hand, and rest without finger movement. The sequence of finger tapping starts with index finger and progressing outward to the little (pinky) finger. Continuous finger tapping motion was required until different condition was presented. During the rest condition, the participants were asked to remain still with their eyes open until the next instruction appears on the display screen.

The motor task is a block-designed test, and the specific sequence of the task blocks (not disclosed to the participants) was counter-balanced over three runs as followed:

Run 1: Rest, Left, Rest, Right, Rest, Left, Rest, Right, Rest
Run 2: Rest, Right, Rest, Left, Rest, Right, Rest, Left, Rest
Run 3: Rest, Left, Rest, Left, Rest, Right, Rest, Right, Rest
Each run contained nine short blocks of 34 s, and the duration of each run was 330.897 s.

### Data analysis

#### Functional MRI data preprocessing

Preprocessing was performed using tools from FSL (FMRIB's Software Library) (Smith et al., 2004), MATLAB (Matrix Laboratory), and toolboxes from SPM (Statistical Parametric Mapping). Skull-stripping on acquired images was conducted through an automated process via Brain Extraction Tool (BET) and later visually inspected for consistency and accuracy. Rigid body motion correction was applied using MCFLIRT, which also allowed absolute and relative mean displacement to be extracted and subsequently included as covariates in statistical analysis. Spatial smoothing was completed using Gaussian kernel of Full-Width-Half-Maximum (FWHM) 6.0 mm. high-pass temporal filtering was applied using a cut-off of 120 s. For the purpose of functional connectivity analysis, an additional low-pass temporal filtering was applied to restrict the fMRI signal fluctuation between 0.008<f<0.080 Hz. The inclusion of a low-pass filter also enables the elimination of high frequency signals that could potentially confound our results. Participants' preprocessed functional data were first registered to each individuals' skull-stripped high resolution T1 anatomical images and then to the standardized 152 T1 Montreal Neurological Institute (MNI) space.

Regression of cerebral-spinal fluid (CSF) signal, white matter signal, global brain signal, and motion parameters were completed to remove both physiological and non-physiological noises as well as excess movement from the data.

#### Independent component analysis (ICA)

Independent component analysis was completed using FSL-MELODIC. Highpass filter cutoff was set at 120 s; motion correction was applied via MCFLIRT; spatial smoothing was performed with 6 mm FWHM Gaussian kernel; preprocessed data was subsequently registered first to the brain-extracted subject-specific high resolution T1 structural images then further registered to a standardized Montreal Neurological Institute (MNI152) brain space template provided within the FSL package. Independent components were estimated through FSL-MELODIC using Laplace approximation to the Bayesian evidence for a probabilistic principal components model (Sala-Llonch et al., 2012). The number of data dimension estimate was set at 20, which was reported to provide sufficient power of detection (Smith et al., 2009; Biswal et al., 2010). The default value of *p* > 0.5 for alternative hypothesis test (an automated threshold that fits a mixture model to the histogram of intensity value) was selected and applied to each component map. The component map outputs were visually inspected to identify relevant neural networks and noise structures.

To examine between group differences, FSL Dual Regression (Beckmann et al., 2009; Filippini et al., 2009) was performed to compare: (1) Normal weight > Overweight, (2) Normal weight > Obese, (3) Overweight > Normal weight, (4) Obese > Normal weight. In FSL Dual Regression, for each subject, the group-average spatial maps are first inputted as spatial-regressors into the subject's 4D dataset. This generates one set of subject-specific time-series data per group-level spatial map. These subject-specific time-series data were then used as temporal-regressors into the same subject's 4D dataset, which outputs one set of subject-specific spatial map per group-level spatial map. Subsequently, group differences are tested using FSL's randomize permutation tool (default setting selected for 5000 permutations).

#### Seed-based functional connectivity analysis

Results from the ICA in the previous section guided our choice of networks to include in the whole brain analysis. Neural networks represented by statistically significant group contrast component maps were selected for seed-based analysis. Seed-selection of the networks of interested was based on our previous work (Hsu et al., 2014) and published literature (Voss et al., 2010).

For all regions of interest (ROI) included in the analysis, preprocessed time-series data were extracted with 14 mm spherical ROI drawn around their respective MNI coordinates in standard space (Voss et al., 2010). The different conditions (i.e., left, right, and rest) within each block of the motor task were temporally spliced and compiled (Fair et al., 2007). In order to perform temporal concatenation of the time-series data, the stimulus onset time for each task condition was acquired from the task program. Volumes of the data were then sorted according to their respective condition. Once the data were properly categorized, the tapping-specific volumes (e.g., all the “left tapping” and “right tapping” volumes) were combined using a bash script provided in the FSL program (i.e., “left tapping” and “right tapping” volumes concatenated into “tapping”). Consequently, two sets of data were produced: “resting” and “tapping”. The first three volumes of any condition were removed to account for delay of the hemodynamic response.

Regions of interest time-series data were cross-correlated with every voxel within the brain to establish functional connections of the associated neural networks. Individual-level within-subject results were generated via ordinary least squares (OLS) in FSL by congregating the voxel-wise functional connectivity maps from each condition. Group-level comparison was performed using a mixed-level OLS analysis. The statistical map thresholding was set at *Z* = 2.33, with cluster correction of *p* < 0.05. Lastly, Pearson's correlation coefficients were calculated between all ROI.

#### Statistical analysis

Statistical analysis was performed on resulting data from the seed-base functional connectivity analysis. To normalize our data, the Pearson's correlation coefficients were converted into Fisher's z correlation coefficients via Fisher's r-to-z transformation (Konishi, 1981) in MATLAB. Fisher's transformation generates normally distributed sample distribution and ensures the variance of the correlation coefficient remain constant for all values in the sample population correlation (Konishi, 1981).

To reduce Type I error and produce more robust signal, the number of comparisons were reduced by calculating the mean correlation of all the ROI-pairs within each of the networks as well as all ROI-pairs between the networks (Hsu et al., 2014). Therefore, for the DMN, there is a total of 15 ROI-pairs based on all possible combination of the ROIs listed in **Table 4**. The mean network correlation for the DMN was calculated by taking the sum of the 15 Fisher's z correlation coefficients and then divided the total value by 15.

Statistical analysis was performed using IBM SPSS 19 for Windows (SPSS Inc., Chicago, IL). Descriptive data are reported for variables of interest. Comparisons of group characteristics at baseline and 12-month were conducted using a Chi Square test for differences in proportions and ANOVAs for differences in means. Analysis of covariance (ANCOVA) was completed to statistically test for significant differences in mean network functional connectivity between: (1) normal BMI and overweight individuals; and (2) normal BMI and obese individuals. In the model, baseline FCI, and mean relative head motion (extracted from McFLIRT) (Power et al., 2012; Van Dijk et al., 2012) were included as covariates. The overall alpha value was set at *p* ≤ 0.05.

**Figure 2:**
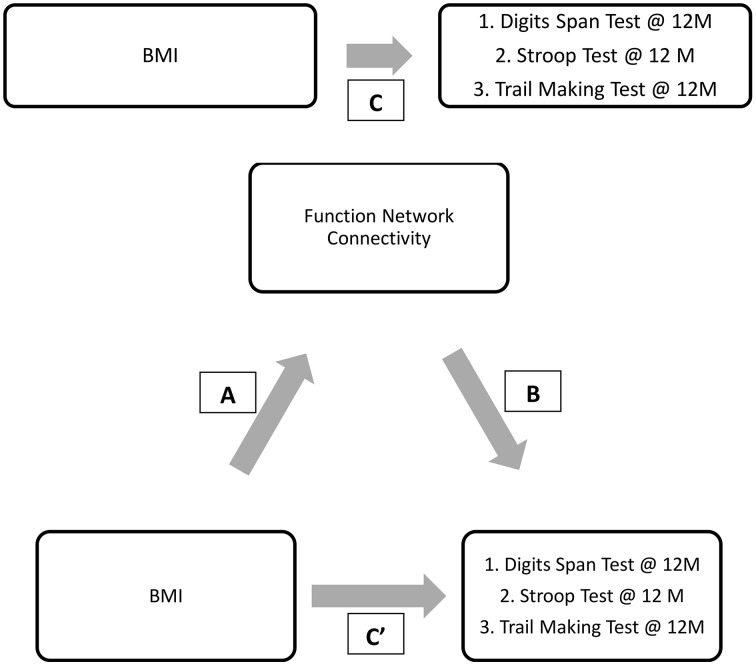
**Proposed mediation models**. “C” represents the total effect; “C”' represent the direct effect; “A and B” in conjunction represents the indirect effect.

Mediation analysis was performed using the PROCESS Macro for SPSS (Hayes, 2012). In the mediation model, BMI was entered as the independent variable; network connectivity of interest (that was significantly different between groups) was entered as the mediator; and the cognitive function measures at 12-month (that was significantly correlated with the network connectivity of interest) as dependent variables (Figure 2). Sex, age, FCI, and baseline MoCA were included as covariates in all models. Sex was included as a covariate as it was statistically different between the groups; while age, FCI, and baseline cognitive performance measure were included due to biological relevance. Two separate linear regression analyses calculated the parameter values for paths A, B, and C' of the proposed mediation model (Figure 2). In the regression analysis for path A, network connectivity was entered as the dependent variable while the covariates were included in the first block of a hierarchical regression analysis. The main predictor—BMI—was entered in the second block of the hierarchical regression analysis, and represents Path A. The second linear regression analysis included both network connectivity and BMI as predictors and cognitive function as the dependent variable. The effect of network connectivity on cognitive function represents path B, and the effect of BMI (once accounting for network connectivity) on cognitive function represents path C'. It is important to note that each model only tested one mediator and one dependent variable at one time. Lastly, we computed the indirect effect (Path A^*^ Path B), which quantifies the size of mediation via network connectivity. Because the indirect effect is non-normally distributed (Hayes and Scharkow, 2013), we calculated the 95% confidence interval (CI) around the indirect effect using 5000 bootstrapped resamples. A 95% CI not containing zero is evidence for a significant indirect effect.

## Results

### Participant demographics

A total of 66 study participants completed both baseline and 12-month assessments. Among the 66 participants, 24 were classified in the normal BMI group, 27 in the overweight BMI group, and 15 in the obese BMI group. Table 1 provides baseline and 12-month descriptive statistics for the three BMI groups. Sex was not evenly distributed across the different BMI groups (Table 1). In the normal and obese BMI groups, there were significantly more female participants compared with male participants; whereas in the overweight group, the distribution of male and female participants was more evenly divided. Compared with the normal BMI group, both overweight, and obese groups had significantly lower mean MoCA scores at baseline. Notably, both the overweight and obese groups had a mean baseline MoCA score suggestive of mild cognitive impairment (Nasreddine et al., 2005). However, at 12-months, this difference in MoCA scores was no longer apparent.

**Table 1:** **Study participant demographics**.

**Variable**	**Baseline normal (*n* = 24)**		**12-M normal (*n* = 24)**		**Baseline overweight (*n* = 27)**		**12-M overweight (*n* = 27)**		**Baseline *p*-value**	**12-M *p*-value**	**Baseline obese (*n* = 15)**		**12-M obese (*n* = 15)**		**Baseline *p*-value**	**12-M *p*-value**
	**Mean**	**SD**	**Mean**	**SD**	**Mean**	**SD**	**Mean**	**SD**	**–**	**–**	**Mean**	**SD**	**Mean**	**SD**	**–**	**–**
Sex (m/f)	3∕21				12∕15				**0.01**		2∕13				0.95	
Height (cm)	162.28	5.90	162.01	5.66	167.44	9.06	165.50	10.60	0.51	0.78	164.47	7.88	164.31	7.91	0.24	0.34
Weight (kg)	58.65	5.17	58.89	5.95	75.79	10.26	75.66	10.41	**<0.01**		88.35	14.73	87.87	14.44	**<0.01**	
BMI	22.29	1.86	22.44	2.07	26.94	1.64	27.60	2.62	**<0.01**		32.67	4.52	32.48	4.46	**<0.01**	
Age (years)	74.33	2.90	75.33	2.90	73.65	3.12	74.96	3.24	0.42	0.67	74.33	2.77	75.15	2.77	1.00	0.85
MoCA (30 pts)	26.04	3.11	25.17	3.83	24.15	2.89	24.25	2.94	**0.04**	0.34	23.33	3.46	23.38	2.66	**0.01**	0.12
MMSE (30 pts)	28.29	1.52	28.25	1.80	28.50	1.36	28.21	1.62	0.63	0.93	28.33	1.80	27.77	1.74	0.94	0.42
Functional comorbidity Index	2.96	2.10	3.08	1.84	2.69	1.41	3.38	1.69	0.64	0.59	3.33	2.64	4.15	2.23	0.57	0.10
Geriatric depression scale	0.58	1.72	0.33	1.13	0.38	0.85	1.08	2.65	0.59	0.18	0.60	1.06	0.54	1.45	0.97	0.76
Digit span forward	8.17	2.62	8.21	1.72	8.11	2.29	8.27	2.31	0.94	0.93	7.67	3.04	7.43	3.03	0.82	0.32
Digit span backward	4.96	2.24	4.67	2.12	4.07	2.02	4.19	2.30	0.15	0.46	3.87	2.36	4.21	2.29	0.13	0.55
Digit span test	3.21	2.70	3.54	1.91	3.88	1.90	4.00	2.54	0.29	0.47	3.80	1.97	3.31	1.97	0.43	0.76
Stroop 3 (s)	106.61	30.61	98.39	23.31	104.31	23.76	101.84	22.02	0.79	0.67	119.28	37.17	118.36	42.42	0.21	**0.04**
Stroop 2 (s)	54.92	13.35	53.27	9.86	57.16	12.48	56.52	8.94	0.55	0.26	57.13	13.65	56.49	11.61	0.62	0.34
Stroop Test (s)	51.69	22.69	45.12	18.94	47.14	18.05	45.62	17.22	0.51	0.92	66.63	33.89	53.45	15.50	0.07	0.18
Trail making B (s)	107.52	50.48	105.83	49.71	104.43	35.82	97.84	26.79	0.80	0.45	100.38	42.91	91.61	23.85	0.63	0.27
Trail Making A (s)	59.05	19.06	58.86	20.23	57.94	10.89	54.19	14.55	0.81	0.37	58.21	21.17	54.16	21.25	0.88	0.45
Trail making Test (s)	48.47	42.54	46.98	39.44	45.77	32.72	41.69	23.94	0.93	0.56	95.84	203.33	38.51	27.22	0.16	0.44

Normal BMI group was used as the reference group for comparison with overweight and obese groups; Digit Span Test score was calculated by taking Digit Span Forward minus Digit Span Backward; Stroop Test score was calculated by taking Stroop 3 minus Stroop 2; Trail Making score was calculated by taking Trail Making B minus Trail Making A. The bold values in the table reflect values that are statistically significant.

Table 2 provides the descriptive statistics for change in cognitive function over the 12-month study period for all three BMI groups. There were no significant differences among the groups in regards to change in cognitive function. However, it is notable that compared with the normal and overweight groups, the obese group showed the greatest improvement in the Digit Span Test performance (22.37% improvement; Table 2).

**Table 2:** **Change in cognitive test performance over 12-months**.

**Variable**	**Normal (*n* = 24)**			**Overweight (*n* = 27)**				**Obese (*n* = 15)**			
	**Mean**	**SD**	**% change**	**Mean**	**SD**	**% change**	***p*-value^a^**	**Mean**	**SD**	**% Change**	***p*-value^a^**
Δ Digit span test	0.33	3.50	10.28	0.12	2.82	3.09	0.91	−0.85	2.88	−22.37%	0.26
Δ Stroop test (s)	−6.57	19.38	−12.71	−1.72	14.27	−3.65	0.32	−3.28	29.79	−4.92%	0.64
Δ Trail making (s)	−1.49	37.91	−3.07	−2.80	36.30	6.12	0.96	1.26	24.25	1.31%	0.61
Δ MoCA	−0.88	2.88	−3.38	0.12	2.98	0.50	0.21	−0.64	1.91	−2.74%	0.80

Δ in performance scores were calculated by taking performance at 12-month minus performance at baseline; negative score and percent change in Δ Digit Span Test, Δ Stroop Test and ΔTrail Making reflect improvement; positive score in Δ MoCA reflects improvement.

a Comparison to normal weight group.

### Functional connectivity of fallers vs. non-fallers

#### Independent component analysis

##### Resting-state

**Figure 3:**
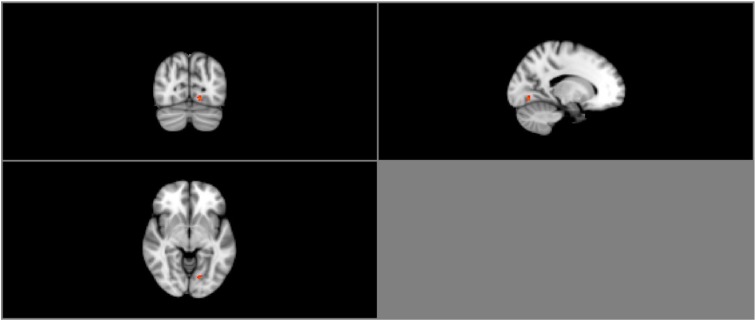
**Resting-state group contrast**. Highlighted areas show regions where Normal > Overweight in FEN functional connectivity (*P* < 0.05) during resting-state.

Group ICA estimation generated a user-designated 25 components. Among the 25 independent components, five components were identified as relevant neural networks through visual inspection (Table 3A; Figure S1 in Supplementary Material). Group comparison of these five components revealed a significant difference between the normal BMI group and overweight group in the right FEN (*p* = 0.05; Figure 3). However, this difference was no longer significant after correcting for multiple comparisons (Bonferroni corrected *p* = 0.005; *p* < 0.05/2groups^*^5components).

**Table 3A:** **Relevant resting-state independent components**.

**Independent components**	**Networks**
Component 4	Visual network
Component 6	Sensori-motor network
Component 7	Default mode network
Component 21	R. Fronto-executive network
Component 22	L. Fronto-executive network

##### Task-state

**Figure 4:**
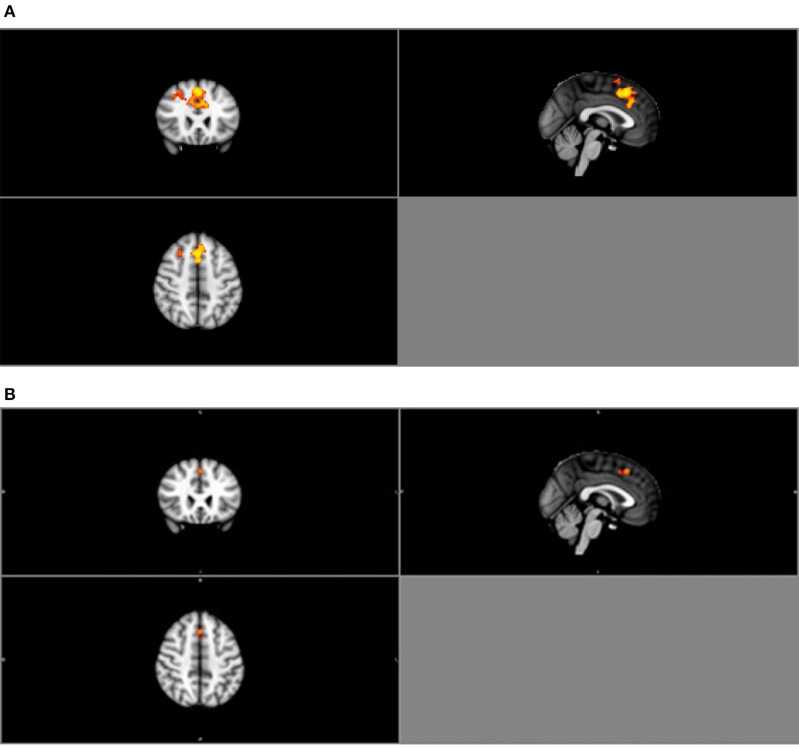
**(A)** Group contrast during finger tapping. Highlighted areas show regions where Normal > Obese in DMN functional connectivity (*P* < 0.05) during finger tapping. **(B)** Bonferroni-corrected group contrast during finger tapping. Highlighted areas show regions where Normal > Obese in DMN functional connectivity (*p* < 0.006) during finger tapping.

Similarly, group ICA estimated a pre-set value of 25 components. Of the 25 components, four were deemed relevant via visual inspection (Table 3B; Figure S2 in Supplementary Material). Group comparison of these six components revealed a significant difference between the normal BMI and obese groups in the DMN (*p* < 0.05; Figure 4A). The between-group difference in the DMN remained significant after adjusting the alpha level for multiple comparison (Bonferroni corrected *p* < 0.006 based on 0.05/2groups^*^4components; Figure 4B).

**Table 3B:** **Relevant task-state independent components**.

**Independent components**	**Networks**
Component 4	Default mode network
Component 9	Sensori-motor network
Component 19	Fronto-executive network
Component 21	Fronto-parietal network

#### Seed-based analysis

In light of the ICA results, only the DMN was considered in the seed-based functional connectivity analysis. Key regions within the DMN selected for the analysis were the posterior cingulate cortex (PCC), ventral and superior frontal medial cortices (FMC), middle temporal gyrus (MTG), parahippocampal gyrus (PHG), middle frontal gyrus (MFG), and lateral occipital cortex (LOC) (Fox et al., 2005; Buckner et al., 2008). Current understanding of the functional roles of the DMN include retrieval of memory from past events, future planning, and self-awareness (Andrews-Hanna et al., 2007; Buckner et al., 2008). Particularly, multiple regions within the DMN are involved in semantic memory and disrupted DMN connectivity correlates with semantic memory impairment (Gardini et al., 2015). Research also suggests that the strength of connection between regions within the DMN positively correlates with working memory performance (Hampson et al., 2006; Sambataro et al., 2010). Moreover, abnormal DMN connectivity pattern is significantly associated with MCI (Greicius, 2008; Qi et al., 2010; Petrella et al., 2011). The respective MNI space coordinates for each region of interest (ROI) are provided in Table 4. Correlation between regions of the DMN was extracted for the purpose of performing partial correlation and mediation statistical analyses.

**Table 4:** **Neural networks and regions of interests included in the analysis**.

**Neural networks**	**Region of interest**	**MNI coordinates (mm)**		
		**X**	**Y**	**Z**
DMN	PCC	8	−56	30
	FMC	−2	54	−12
	RMTG	58	−10	−18
	LMTG	−52	−14	−20
	RPHG	24	−26	−20
	LPHG	−26	−24	−20
	LMFG	−30	20	50
	RLOC	54	−62	32
	LLOC	−44	−72	30

PCC, posterior cingulate cortex; FMC, frontal medial cortex; RMTG/LMTG, right/left middle temporal gyrus; RPHG/LPHG, right/left parahippocampal gyrus; LMFG, left middle frontal gyrus; RLOC/LLOC, right/left parietal cortex.

##### Correlation results

We found higher baseline BMI was associated with less DMN connectivity at baseline (partial *r* = −0.45, *p* = 0.001), but did not predict performance on any of the 12-month cognitive measures (see Table 5A). Additionally, connectivity of the DMN during finger tapping at baseline was significantly associated with Digit Span Test performance at 12-month, independent of the covariates and baseline Digit Span Test performance (partial *r* = 0.30, *p* = 0.02; Table 5B). Specifically, higher DMN connectivity was associated with higher digit span test scores (i.e., poorer working memory).

**Table 5A:** **Partial correlations of baseline BMI, network connectivity, and cognitive outcome measures**.

**Variables**	**DMN finger tapping**		**Digit span test 12-month**		**Stroop test 12-month**		**Trail making test 12-month**	
	**Partial *r***	***p*-value**	**Partial *r***	***p*-value**	**Partial *r***	***p*-value**	**Partial *r***	***p*-value**
Baseline BMI	−0.45	**0.001**	−0.001	0.99	0.22	0.10	−0.001	0.99

Controlled for baseline FCI, baseline age, sex, and baseline cognitive performance. Digit Span Test score was calculated by taking Digit Span Forward minus Digit Span Backward; Stroop Test score was calculated by taking Stroop 3 minus Stroop 2; Trail Making score was calculated by taking Trail Making B minus Trail Making A. The bold value in the table reflect p-value that is statistically significant.

**Table 5B:** **Partial correlations of connectivity and cognitive outcome measures**.

**Variables**	**DMN connectivity during finger tapping**	
	**Partial *r***	***p*-value**
Digit span test at 12-month	0.30	**0.02**
Stroop test at 12-month	−0.02	0.87
Trail making test at 12-month	0.15	0.25

Controlled for baseline FCI, baseline age, sex, and baseline cognitive performance. Digit Span Test score was calculated by taking Digit Span Forward minus Digit Span Backward; Stroop Test score was calculated by taking Stroop 3 minus Stroop 2; Trail Making score was calculated by taking Trail Making B minus Trail Making A. The bold value in the table reflect p-value that is statistically significant.

#### Mediation analyses

**Figure 5:**
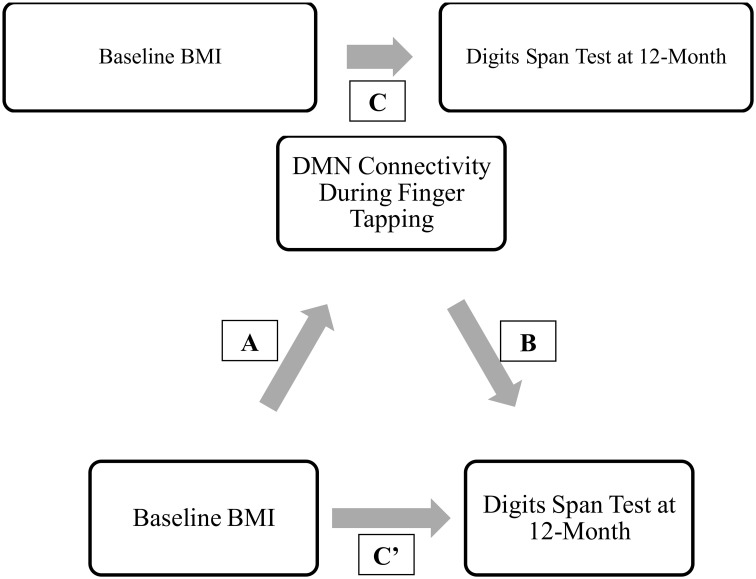
**Final proposed mediation model**. ‘C” represents the total effect; “C”’ represent the direct effect; “A and B” in conjunction represents the indirect effect.

Given the preceding evidence that baseline BMI is significantly associated with DMN connectivity and that DMN connectivity predicts verbal working memory (digit span test performance) at 12-month, we conducted a mediation analysis shown in Figure 5.

**Figure 6:**
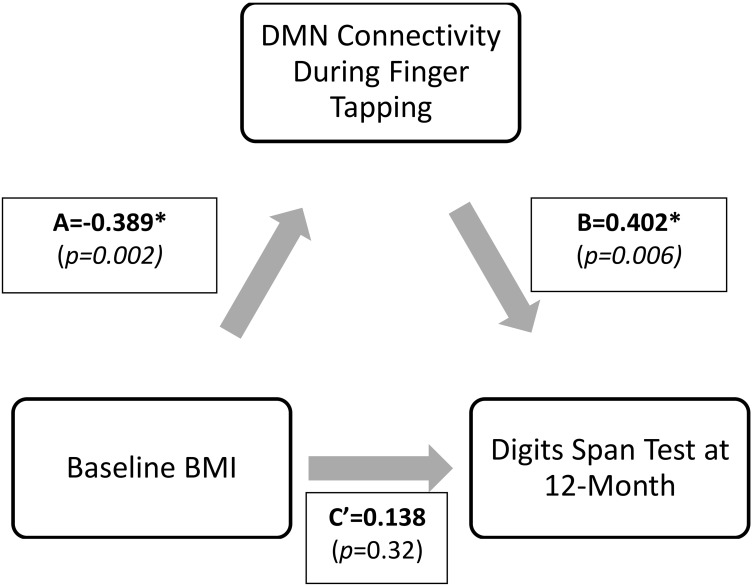
**Direct and indirect effects of the proposed mediation model**. ^*^Digit Span Test score is calculated as Digit Span Forward-Backward; therefore, lower value describes higher performance. The indirect effect (Path A^*^ Path B) was −0.155, 95%CI (−0.313, −0.053). Standardized coefficients (β) are shown.

Results from the mediation analysis suggest that DMN connectivity during finger tapping significantly mediated the relationship between baseline BMI and Digit Span Test at 12-month (indirect effect: −0.155, 95% CI [−0.313, −0.053]). In light of the fact that the total effect of BMI on Digit Span Test performance at 12-month (Path C) was not significant (β = −0.02, *p* = 0.90), this significant indirect effect suggests that decreased task-related DMN connectivity is a pathway by which obese older adults maintain working memory performance over time equal to their lean counterparts. Detailed results are shown in Figure 6.

## Discussion

At baseline, we found a significant difference in functional connectivity within the DMN between obese older adults and their normal weight peers. Specifically, obese older adults showed lower DMN connectivity during finger tapping as compared with those with normal BMI. Moreover, lower task-related connectivity in the DMN at baseline was significantly associated with better working memory at 12-months. Finally, our mediation analysis revealed that lower DMN connectivity may an underlying mechanism by which higher BMI is associated with better cognitive performance among older adults. That is, we found a significant indirect effect from baseline BMI to DMN connectivity to 12-month working memory. To our knowledge, this is the first study to explore the functional neural correlates by which higher BMI might be neuroprotective among older adults without frank dementia.

Our mediation results concur and extend existing literature. As described previously, DMN is susceptible to age-related disruption and is associated with a broad range of neuropsychological or neurodegenerative diseases (Buckner et al., 2008; Greicius, 2008). Often discussed as a task-negative network, the DMN is characterized by task-induced deactivation, a process that is believed to be related to allocation of neural resources relative to task demand (Hu et al., 2013). Although the connectivity of regions within this network remains during task-state, the strength of these region-to-region connections is attenuated (Fransson, 2006). Thus, greater task-related DMN deactivation may indicate better DMN integrity.

Why might a higher BMI be associated with greater DMN integrity in older adults? While more research is needed in this area, there is evidence to suggest amyloid deposition as a contributing factor. First, in a cross-sectional analysis of data from the Alzheimer's Disease Neuroimaging Initiative, Vidoni et al. (2011) found that overweight non-demented older adults had higher CSF ß-amyloid, less CSF tau and global PiB uptake than their normal weight counterparts. These results suggest that lower BMI among older adults without cognitive or functional impairment is associated with higher AD pathophysiology. Second, Mormino et al. (2011) showed that amyloid deposition spatially overlaps the DMN, thereby inducing aberrant DMN connectivity patterns (Sperling et al., 2009; Koch et al., 2014). Thus, higher BMI may be associated with DMN integrity because it is a biomarker of reduced AD pathophysiology, which has shown to directly impact the DMN.

Our data also suggest that the association between BMI and cognitive function may vary between cross-sectional observations and prospective observations, and thus, contribute to the current uncertainty in the literature. Specifically, we observed a significant difference between the three BMI groups at baseline in MoCA performance. However, this difference was no longer apparent 12-months later. Descriptively, over the 12-month study period we observed a one point decline in the mean MOCA score for among older adults with normal BMI while those who were overweight demonstrated maintenance of MoCA performance. Thus, our prospective results align with previous evidence that suggests low BMI is associated with greater cognitive decline over time (Grundman et al., 1996; Hu et al., 2002; Han et al., 2009). For example, Han et al. (2009) reported that increased body weight over time in older men who were initially obese at baseline was associated with improved cognitive function, whereas reduced waist-to-hip ratio over time was associated with reduced cognitive function.

There are several limitations that should be considered. First, we were unable to explore other biological and genetic factors, such as white matter lesion volumes, ß-amyloid, tau, and ApoE4 genotype, which could potentially also affect brain functional connectivity. Also, between-group differences in functional connectivity may be dependent on the specific task imposed. Thus, the generalizability of our findings to other task states will be an important direction of future research. Recruitment method for study participants was entirely volunteer-based; hence our study participants were likely more motivated and thus may not be representative of the community-dwelling older adults. In addition, our study sample consisted exclusively of older adults without significant cognitive or physical impairments; therefore our results may not be generalizable beyond the current population. Moreover, BMI may not accurately reflect obesity. For example, in individuals with greater muscle mass, higher BMI is not representative of elevated percent body fat. In terms of the neuroimaging procedure, the design of our motor task (interleaving blocks of finger tapping and rest) may have reduced our ability to accurately investigate the functional network connectivity during rest as neural activity during the finger tapping block may “bleed” into the immediate subsequent resting block. Lastly, we had a relatively small sample size of 66 individuals, which limits our power to detect small effects. Thus, future studies with larger samples sizes are required to confirm our current findings.

## Conclusion

Obesity is an increasingly prevalent global healthcare issue due to its clinical relevance to various clinical conditions including cardiovascular diseases and dementia. Current knowledge in obesity-related research identified elevated BMI is correlated with cognitive decline in younger adults; nevertheless, the relationship between increased BMI and cognitive function remain equivocal in older adults. Our study presents novel evidence that higher BMI may be cognitive beneficial in the older adult population through a mediated relationship with brain function, and contributes to our current understanding of the interplay between BMI and cognitive function in late life.

## Funding

TA is a Canada Research Chair (Tier 2) in Physical Activity, Mobility, and Cognitive Neuroscience. CH is an Alzheimer's Society Research Program Doctoral trainee. JB is a Canadian Institutes of Health Research and Michael Smith Foundation for Health Research postdoctoral fellow. This work was supported by the Canadian Institute of Health Research (MOB-93373) to TA.

### Conflict of interest statement

The authors declare that the research was conducted in the absence of any commercial or financial relationships that could be construed as a potential conflict of interest.

## References

[B1] Andrews-Hanna J. R. Snyder A. Z. Vincent J. L. Lustig C. Head D. Raichle M. E. (2007). Disruption of large-scale brain systems in advanced aging. Neuron 56, 924–935. 10.1016/j.neuron.2007.10.038 18054866 PMC2709284

[B2] Arnold G. Boone K. B. Lu P. Dean A. Wen J. Nitch S.. (2005). Sensitivity and specificity of finger tapping test scores for the detection of suspect effort. Clin. Neuropsychol. 19, 105–120. 10.1080/13854040490888567 15814482

[B3] Artham S. M. Lavie C. J. Milani R. V. Ventura H. O. (2008). The obesity paradox: impact of obesity on the prevalence and prognosis of cardiovascular diseases. Postgrad. Med. 120, 34–41. 10.3810/pgm.2008.07.1788 18654066

[B4] Bandeen-Roche K. Xue Q.-L. Ferrucci L. Walston J. Guralnik J. M. Chaves P.. (2006). Phenotype of frailty: characterization in the women's health and aging studies. J. Gerontol. A Biol. Sci. Med. Sci. 61A, 262–266. 10.1093/gerona/61.3.262 16567375

[B5] Beckmann C. F. Mackay C. E. Filippini N. Smith S. M. (2009). Group comparison of resting-state FMRI data using multi-subject ICA and dual regression. Neuroimage 47, S148 10.1016/S1053-8119(09)71511-3

[B6] Biswal B. B. Mennes M. Zuo X. N. Gohel S. Kelly C. Smith S. M.. (2010). Toward discovery science of human brain function. Proc. Natl. Acad. Sci. USA. 107, 4734–4739. 10.1073/pnas.0911855107 20176931 PMC2842060

[B7] Bostick R. M. Potter J. D. Kushi L. H. Sellers T. A. Steinmetz K. A. Mckenzie D. R.. (1994). Sugar, meat, and fat intake, and non-dietary risk factors for colon cancer incidence in Iowa women (United States). Cancer Causes Control 5, 38–52. 10.1007/BF01830725 8123778

[B8] Boxer A. L. Rankin K. P. Miller B. L. Schuff N. Weiner M. Gorno-Tempini M. L.. (2003). Cinguloparietal atrophy distinguishes Alzheimer disease from semantic dementia. Arch. Neurol. 60, 949–956. 10.1001/archneur.60.7.949 12873851

[B9] Buchman A. S. Wilson R. S. Bienias J. L. Shah R. C. Evans D. A. Bennett D. A. (2005). Change in body mass index and risk of incident Alzheimer disease. Neurology 65, 892–897. 10.1212/01.wnl.0000176061.33817.90 16186530

[B10] Buckner R. L. Andrews-Hanna J. R. Schacter D. L. (2008). The brain's default network: anatomy, function, and relevance to disease. Ann. N.Y. Acad. Sci. 1124, 1–38. 10.1196/annals.1440.011 18400922

[B11] Campbell K. L. Grady C. L. Ng C. Hasher L. (2012). Age differences in the frontoparietal cognitive control network: implications for distractibility. Neuropsychologia 50, 2212–2223. 10.1016/j.neuropsychologia.2012.05.025 22659108 PMC4898951

[B12] Childers D. K. Allison D. B. (2010). The ‘obesity paradox’: a parsimonious explanation for relations among obesity, mortality rate and aging? Int. J. Obes. (Lond.) 34, 1231–1238. 10.1038/ijo.2010.71 20440298 PMC3186057

[B13] Chute C. G. Willett W. C. Colditz G. A. Stampfer M. J. Baron J. A. Rosner B.. (1991). A prospective study of body mass, height, and smoking on the risk of colorectal cancer in women. Cancer Causes Control 2, 117–124. 10.1007/BF00053131 1873436

[B14] Cockrell J. R. Folstein M. F. (1988). Mini-Mental State Examination (MMSE). Psychopharmacol. Bull. 24, 689–692. 3249771

[B15] Dosenbach N. U. Visscher K. M. Palmer E. D. Miezin F. M. Wenger K. K. Kang H. C.. (2006). A core system for the implementation of task sets. Neuron 50, 799–812. 10.1016/j.neuron.2006.04.031 16731517 PMC3621133

[B16] Dyer A. R. Elliott P. (1989). The INTERSALT study: relations of body mass index to blood pressure. INTERSALT Co-operative Research Group. J. Hum. Hypertens. 3, 299–308. 2810326

[B17] El Fakhri G. Kijewski M. F. Johnson K. A. Syrkin G. Killiany R. J. Becker J. A.. (2003). MRI-guided SPECT perfusion measures and volumetric MRI in prodromal Alzheimer disease. Arch. Neurol. 60, 1066–1072. 10.1001/archneur.60.8.1066 12925361

[B18] Elias M. F. Elias P. K. Sullivan L. M. Wolf P. A. D'agostino R. B. (2003). Lower cognitive function in the presence of obesity and hypertension: the Framingham heart study. Int. J. Obes. Relat. Metab. Disord. 27, 260–268. 10.1038/sj.ijo.802225 12587008

[B19] Fair D. A. Schlaggar B. L. Cohen A. L. Miezin F. M. Dosenbach N. U. Wenger K. K.. (2007). A method for using blocked and event-related fMRI data to study “resting state” functional connectivity. Neuroimage 35, 396–405. 10.1016/j.neuroimage.2006.11.051 17239622 PMC2563954

[B20] Filippini N. Macintosh B. J. Hough M. G. Goodwin G. M. Frisoni G. B. Smith S. M.. (2009). Distinct patterns of brain activity in young carriers of the APOE-epsilon4 allele. Proc. Natl. Acad. Sci. U.S.A. 106, 7209–7214. 10.1073/pnas.0811879106 19357304 PMC2678478

[B21] Flegal K. M. Kit B. K. Orpana H. Graubard B. I. (2013). Association of all-cause mortality with overweight and obesity using standard body mass index categories: a systematic review and meta-analysis. JAMA 309, 71–82. 10.1001/jama.2012.113905 23280227 PMC4855514

[B22] Fogassi L. Luppino G. (2005). Motor functions of the parietal lobe. Curr. Opin. Neurobiol. 15, 626–631. 10.1016/j.conb.2005.10.015 16271458

[B23] Fox M. D. Snyder A. Z. Vincent J. L. Corbetta M. Van Essen D. C. Raichle M. E. (2005). The human brain is intrinsically organized into dynamic, anticorrelated functional networks. Proc. Natl. Acad. Sci. U.S.A. 102, 9673–9678. 10.1073/pnas.0504136102 15976020 PMC1157105

[B24] Fransson P. (2006). How default is the default mode of brain function? Further evidence from intrinsic BOLD signal fluctuations. Neuropsychologia 44, 2836–2845. 10.1016/j.neuropsychologia.2006.06.017 16879844

[B25] Gardini S. Venneri A. Sambataro F. Cuetos F. Fasano F. Marchi M.. (2015). Increased functional connectivity in the default mode network in mild cognitive impairment: a maladaptive compensatory mechanism associated with poor semantic memory performance. J. Alzheimers Dis. 45, 457–470. 10.3233/JAD-142547 25547636

[B26] Grady C. L. Protzner A. B. Kovacevic N. Strother S. C. Afshin-Pour B. Wojtowicz M.. (2010). A multivariate analysis of age-related differences in default mode and task-positive networks across multiple cognitive domains. Cereb. Cortex 20, 1432–1447. 10.1093/cercor/bhp207 19789183 PMC3181214

[B27] Graf P. Uttl B. Tuokko H. (1995). Color- and picture-word Stroop tests: performance changes in old age. J. Clin. Exp. Neuropsychol. 17, 390–415. 10.1080/01688639508405132 7650102

[B28] Greicius M. (2008). Resting-state functional connectivity in neuropsychiatric disorders. Curr. Opin. Neurol. 21, 424–430. 10.1097/WCO.0b013e328306f2c5 18607202

[B29] Greicius M. D. Srivastava G. Reiss A. L. Menon V. (2004). Default-mode network activity distinguishes Alzheimer's disease from healthy aging: evidence from functional MRI. Proc. Natl. Acad. Sci. U.S.A. 101, 4637–4642. 10.1073/pnas.0308627101 15070770 PMC384799

[B30] Groll D. L. To T. Bombardier C. Wright J. G. (2005). The development of a comorbidity index with physical function as the outcome. J. Clin. Epidemiol. 58, 595–602. 10.1016/j.jclinepi.2004.10.018 15878473

[B31] Grundman M. Corey-Bloom J. Jernigan T. Archibald S. Thal L. J. (1996). Low body weight in Alzheimer's disease is associated with mesial temporal cortex atrophy. Neurology 46, 1585–1591. 10.1212/WNL.46.6.1585 8649553

[B32] Gunstad J. Paul R. H. Cohen R. A. Tate D. F. Spitznagel M. B. Gordon E. (2007). Elevated body mass index is associated with executive dysfunction in otherwise healthy adults. Compr. Psychiatry 48, 57–61. 10.1016/j.comppsych.2006.05.001 17145283

[B33] Gupta A. Mayer E. A. Sanmiguel C. P. Van Horn J. D. Woodworth D. Ellingson B. M.. (2015). Patterns of brain structural connectivity differentiate normal weight from overweight subjects. Neuroimage Clin. 7, 506–517. 10.1016/j.nicl.2015.01.005 25737959 PMC4338207

[B34] Hampson M. Driesen N. R. Skudlarski P. Gore J. C. Constable R. T. (2006). Brain connectivity related to working memory performance. J. Neurosci. 26, 13338–13343. 10.1523/JNEUROSCI.3408-06.2006 17182784 PMC2677699

[B35] Hampson M. Olson I. R. Leung H. C. Skudlarski P. Gore J. C. (2004). Changes in functional connectivity of human MT/V5 with visual motion input. Neuroreport 15, 1315–1319. 10.1097/01.wnr.0000129997.95055.15 15167557

[B36] Han C. Jo S. A. Seo J. A. Kim B. G. Kim N. H. Jo I.. (2009). Adiposity parameters and cognitive function in the elderly: application of “Jolly Fat” hypothesis to cognition. Arch. Gerontol. Geriatr. 49, e133–e138. 10.1016/j.archger.2008.11.005 19108905

[B37] Hao X. Xu D. Bansal R. Dong Z. Liu J. Wang Z.. (2013). Multimodal magnetic resonance imaging: the coordinated use of multiple, mutually informative probes to understand brain structure and function. Hum. Brain Mapp. 34, 253–271. 10.1002/hbm.21440 22076792 PMC4284056

[B39] Hayes A. F. (2012). PROCESS: A Versatile Computational Tool for Observed Variable Mediation, Moderation, and Conditional Process Modeling [White paper]. Available online at: http://www.afhayes.com/public/process2012.pdf

[B38] Hayes A. F. Scharkow M. (2013). The relative trustworthiness of inferential tests of the indirect effect in statistical mediation analysis: does method really matter? Psychol. Sci. 24, 1918–1927. 10.1177/0956797613480187 23955356

[B40] Hayes S. M. Alosco M. L. Forman D. E. (2014). The effects of aerobic exercise on cognitive and neural decline in aging and cardiovascular disease. Curr. Geriatr. Rep. 3, 282–290. 10.1007/s13670-014-0101-x 25750853 PMC4349343

[B41] Hsu C. L. Voss M. W. Handy T. C. Davis J. C. Nagamatsu L. S. Chan A.. (2014). Disruptions in brain networks of older fallers are associated with subsequent cognitive decline: a 12-month prospective exploratory study. PLoS ONE 9:e93673. 10.1371/journal.pone.0093673 24699668 PMC3977422

[B42] Hu X. Okamura N. Arai H. Higuchi M. Maruyama M. Itoh M.. (2002). Neuroanatomical correlates of low body weight in Alzheimer's disease: a PET study. Prog. Neuropsychopharmacol. Biol. Psychiatry 26, 1285–1289. 10.1016/S0278-5846(02)00291-9 12502015

[B43] Hu Y. Chen X. Gu H. Yang Y. (2013). Resting-state glutamate and GABA concentrations predict task-induced deactivation in the default mode network. J. Neurosci. 33, 18566–18573. 10.1523/JNEUROSCI.1973-13.2013 24259578 PMC3834059

[B44] Hubert H. B. Feinleib M. Mcnamara P. M. Castelli W. P. (1983). Obesity as an independent risk factor for cardiovascular disease: a 26-year follow-up of participants in the framingham heart study. Circulation 67, 968–977. 10.1161/01.CIR.67.5.968 6219830

[B45] Koch K. Myers N. E. Göttler J. Pasquini L. Grimmer T. Förster S.. (2014). Disrupted intrinsic networks link amyloid-beta pathology and impaired cognition in prodromal Alzheimer's disease. Cereb. Cortex. [Epub ahead of print]. 10.1093/cercor/bhu151 24996404 PMC4635914

[B46] Konishi S. (1981). Normalizing transformations of some statistics in multivariate analysis. Biometrika 68, 647–651. 10.1093/biomet/68.3.647

[B47] Kuczmarski R. J. Carroll M. D. Flegal K. M. Troiano R. P. (1997). Varying body mass index cutoff points to describe overweight prevalence among U.S. adults: NHANES III (1988 to 1994). Obes. Res. 5, 542–548. 10.1002/j.1550-8528.1997.tb00575.x 9449138

[B48] Mcfadden K. L. Cornier M. A. Melanson E. L. Bechtell J. L. Tregellas J. R. (2013). Effects of exercise on resting-state default mode and salience network activity in overweight/obese adults. Neuroreport 24, 866–871. 10.1097/WNR.0000000000000013 24022176 PMC3937986

[B49] Medalie J. H. Papier C. Herman J. B. Goldbourt U. Tamir S. Neufeld H. N.. (1974). Diabetes mellitus among 10,000 adult men. I. Five-year incidence and associated variables. Isr. J. Med. Sci. 10, 681–697. 4851353

[B50] Millman R. P. Carlisle C. C. Mcgarvey S. T. Eveloff S. E. Levinson P. D. (1995). Body fat distribution and sleep apnea severity in women. Chest 107, 362–366. 10.1378/chest.107.2.362 7842762

[B51] Minoshima S. Foster N. L. Kuhl D. E. (1994). Posterior cingulate cortex in Alzheimer's disease. Lancet 344, 895. 10.1016/S0140-6736(94)92871-1 7916431

[B52] Minoshima S. Giordani B. Berent S. Frey K. A. Foster N. L. Kuhl D. E. (1997). Metabolic reduction in the posterior cingulate cortex in very early Alzheimer's disease. Ann. Neurol. 42, 85–94. 10.1002/ana.410420114 9225689

[B53] Mormino E. C. Smiljic A. Hayenga A. O. Onami S. H. Greicius M. D. Rabinovici G. D.. (2011). Relationships between beta-amyloid and functional connectivity in different components of the default mode network in aging. Cereb. Cortex 21, 2399–2407. 10.1093/cercor/bhr025 21383234 PMC3169663

[B54] Morris J. C. (2005). Early-stage and preclinical Alzheimer disease. Alzheimer Dis. Assoc. Disord. 19, 163–165. 10.1097/01.wad.0000184005.22611.cc 16118535

[B55] Nasreddine Z. S. Phillips N. A. Bédirian V. Charbonneau S. Whitehead V. Collin I.. (2005). The Montreal Cognitive Assessment, MoCA: a brief screening tool for mild cognitive impairment. J. Am. Geriatr. Soc. 53, 695–699. 10.1111/j.1532-5415.2005.53221.x 15817019

[B56] Nilsson L. G. Nilsson E. (2009). Overweight and cognition. Scand. J. Psychol. 50, 660–667. 10.1111/j.1467-9450.2009.00777.x 19930267

[B57] O'Bryant S. E. Humphreys J. D. Smith G. E. Ivnik R. J. Graff-Radford N. R. Petersen R. C.. (2008). Detecting dementia with the mini-mental state examination in highly educated individuals. Arch. Neurol. 65, 963–967. 10.1001/archneur.65.7.963 18625866 PMC2587038

[B58] Ogden C. L. Carroll M. D. Kit B. K. Flegal K. M. (2014). Prevalence of childhood and adult obesity in the United States, 2011-2012. JAMA 311, 806–814. 10.1001/jama.2014.732 24570244 PMC4770258

[B59] Oldfield R. C. (1971). The assessment and analysis of handedness: the Edinburgh inventory. Neuropsychologia 9, 97–113. 10.1016/0028-3932(71)90067-4 5146491

[B60] Park D. C. Reuter-Lorenz P. (2009). The adaptive brain: aging and neurocognitive scaffolding. Annu. Rev. Psychol. 60, 173–196. 10.1146/annurev.psych.59.103006.093656 19035823 PMC3359129

[B61] Persson J. Nyberg L. Lind J. Larsson A. Nilsson L. G. Ingvar M.. (2006). Structure-function correlates of cognitive decline in aging. Cereb. Cortex 16, 907–915. 10.1093/cercor/bhj036 16162855

[B62] Petrella J. R. Sheldon F. C. Prince S. E. Calhoun V. D. Doraiswamy P. M. (2011). Default mode network connectivity in stable vs progressive mild cognitive impairment. Neurology 76, 511–517. 10.1212/WNL.0b013e31820af94e 21228297 PMC3053179

[B63] Power J. D. Barnes K. A. Snyder A. Z. Schlaggar B. L. Petersen S. E. (2012). Spurious but systematic correlations in functional connectivity MRI networks arise from subject motion. Neuroimage 59, 2142–2154. 10.1016/j.neuroimage.2011.10.018 22019881 PMC3254728

[B64] Qi Z. Wu X. Wang Z. Zhang N. Dong H. Yao L.. (2010). Impairment and compensation coexist in amnestic MCI default mode network. Neuroimage 50, 48–55. 10.1016/j.neuroimage.2009.12.025 20006713

[B65] Reuter-Lorenz P. A. Lustig C. (2005). Brain aging: reorganizing discoveries about the aging mind. Curr. Opin. Neurobiol. 15, 245–251. 10.1016/j.conb.2005.03.016 15831410

[B66] Rockwood K. Andrew M. Mitnitski A. (2007). A comparison of two approaches to measuring frailty in elderly people. J. Gerontol. A Biol. Sci. Med. Sci. 62A, 738–743. 10.1093/gerona/62.7.738 17634321

[B67] Sabia S. Kivimaki M. Shipley M. J. Marmot M. G. Singh-Manoux A. (2009). Body mass index over the adult life course and cognition in late midlife: the Whitehall II Cohort Study. Am. J. Clin. Nutr. 89, 601–607. 10.3945/ajcn.2008.26482 19073790 PMC2714395

[B68] Sala-Llonch R. Peña-Gómez C. Arenaza-Urquijo E. M. Vidal-Pineiró D. Bargallo N. Junqué C.. (2012). Brain connectivity during resting state and subsequent working memory task predicts behavioural performance. Cortex 48, 1187–1196. 10.1016/j.cortex.2011.07.006 21872853

[B69] Sambataro F. Murty V. P. Callicott J. H. Tan H. Y. Das S. Weinberger D. R.. (2010). Age-related alterations in default mode network: impact on working memory performance. Neurobiol. Aging 31, 839–852. 10.1016/j.neurobiolaging.2008.05.022 18674847 PMC2842461

[B70] Seeley W. W. Menon V. Schatzberg A. F. Keller J. Glover G. H. Kenna H.. (2007). Dissociable intrinsic connectivity networks for salience processing and executive control. J. Neurosci. 27, 2349–2356. 10.1523/JNEUROSCI.5587-06.2007 17329432 PMC2680293

[B71] Smith S. M. Fox P. T. Miller K. L. Glahn D. C. Fox P. M. Mackay C. E.. (2009). Correspondence of the brain's functional architecture during activation and rest. Proc. Natl. Acad. Sci. U.S.A. 106, 13040–13045. 10.1073/pnas.0905267106 19620724 PMC2722273

[B72] Smith S. M. Jenkinson M. Woolrich M. W. Beckmann C. F. Behrens T. E. Johansen-Berg H.. (2004). Advances in functional and structural MR image analysis and implementation as FSL. Neuroimage 23(Suppl. 1), S208–S219. 10.1016/j.neuroimage.2004.07.051 15501092

[B73] Sperling R. A. Laviolette P. S. O'Keefe K. O'brien J. Rentz D. M. Pihlajamaki M.. (2009). Amyloid deposition is associated with impaired default network function in older persons without dementia. Neuron 63, 178–188. 10.1016/j.neuron.2009.07.003 19640477 PMC2738994

[B74] Spreen O. Strauss E. (1998). A Compendium of Neurological Tests, 2nd Edn. New York, NY: Oxford University Press, Inc.

[B75] Spreng R. N. Stevens W. D. Chamberlain J. P. Gilmore A. W. Schacter D. L. (2010). Default network activity, coupled with the frontoparietal control network, supports goal-directed cognition. Neuroimage 53, 303–317. 10.1016/j.neuroimage.2010.06.016 20600998 PMC2914129

[B76] Sridharan D. Levitin D. J. Menon V. (2008). A critical role for the right fronto-insular cortex in switching between central-executive and default-mode networks. Proc. Natl. Acad. Sci. U.S.A. 105, 12569–12574. 10.1073/pnas.0800005105 18723676 PMC2527952

[B77] Van Den Berg E. Biessels G. J. De Craen A. J. Gussekloo J. Westendorp R. G. (2007). The metabolic syndrome is associated with decelerated cognitive decline in the oldest old. Neurology 69, 979–985. 10.1212/01.wnl.0000271381.30143.75 17785666

[B78] Van Dijk K. R. Sabuncu M. R. Buckner R. L. (2012). The influence of head motion on intrinsic functional connectivity MRI. Neuroimage 59, 431–438. 10.1016/j.neuroimage.2011.07.044 21810475 PMC3683830

[B79] Van Marwijk H. W. Wallace P. De Bock G. H. Hermans J. Kaptein A. A. Mulder J. D. (1995). Evaluation of the feasibility, reliability and diagnostic value of shortened versions of the geriatric depression scale. Br. J. Gen. Pract. 45, 195–199. 7612321 PMC1239201

[B80] Vidoni E. D. Thomas G. P. Honea R. A. Loskutova N. Burns J. M. (2012). Evidence of altered corticomotor system connectivity in early-stage Alzheimer's disease. J. Neurol. Phys. Ther. 36, 8–16. 10.1097/NPT.0b013e3182462ea6 22333920 PMC3288781

[B81] Vidoni E. D. Townley R. A. Honea R. A. Burns J. M. (2011). Alzheimer disease biomarkers are associated with body mass index. Neurology 77, 1913–1920. 10.1212/WNL.0b013e318238eec1 22105948 PMC3233188

[B82] Voss M. W. Prakash R. S. Erickson K. I. Basak C. Chaddock L. Kim J. S.. (2010). Plasticity of brain networks in a randomized intervention trial of exercise training in older adults. Front. Aging Neurosci. 2:32. 10.3389/fnagi.2010.00032 20890449 PMC2947936

[B83] Wang G. J. Volkow N. D. Logan J. Pappas N. R. Wong C. T. Zhu W.. (2001). Brain dopamine and obesity. Lancet 357, 354–357. 10.1016/S0140-6736(00)03643-6 11210998

[B84] Wechsler D. (1981). Wechsler Adult Intelligence Scale - Revised. San Antonio, TX: The Psychological Corporation; Harcourt Brace Jovanovich.

[B85] WHO (1995). Physical Status: The Use and Interpretation of Anthropometry. Report of a WHO Expert Committee. Geneva: World Health Organization. (Technical Report Series, No. 854). 8594834

[B86] WHO (2000). Obesity: Preventing and Managing the Global Epidemic: Report on a WHO Consultation. Geneva: World Health Organization. (WHO Technical Report Series 894). 11234459

[B87] Wijngaarden M. A. Veer I. M. Rombouts S. A. van Buchem M. A. Willems van Dijk K. Pijl H.. (2015). Obesity is marked by distinct functional connectivity in brain networks involved in food reward and salience. Behav. Brain Res. 287, 127–134. 10.1016/j.bbr.2015.03.016 25779924

[B88] Yesavage J. A. Brink T. L. Rose T. L. Lum O. Huang V. Adey M.. (1982). Development and validation of a geriatric depression screening scale: a preliminary report. J. Psychiatr. Res. 17, 37–49. 10.1016/0022-3956(82)90033-4 7183759

[B89] Yesavage J. A. (1988). Geriatric Depression Scale. Psychopharmacol. Bull. 24, 709–711. 3249773

