# Supplementary material for: Elevated body mass index and maintenance of cognitive function in late life: exploring underlying neural mechanisms
Source: Front Aging Neurosci. 2015 Aug 18;7:155. doi: 10.3389/fnagi.2015.00155 (PMC4539697; doi:10.3389/fnagi.2015.00155)
Supplement: Supplementary file 1 [file Image1.PDF]

Supplementary Material

Elevated Body Mass Index and Maintenance of Cognitive Function in Late Life: Exploring Underlying Neural Mechanisms

Chun Liang Hsu [1,2,3,4], Michelle W. Voss [5,6], John Best[1,2,3,4], Todd C. Handy [7], Kenneth Madden [8], Niousha Bolandzadeh [1,2,3,4], Teresa Liu-Ambrose [1,2,3,4]*

[1] Aging, Mobility, and Cognitive Neuroscience Lab, University of British Columbia, Vancouver, British Columbia, Canada
[2] Vancouver Health Research Institute, Vancouver, British Columbia, Canada
[3] Djavad Mowafaghian Center for Brain Health, University of British Columbia, Vancouver, British Columbia, Canada
[4] Center for Hip Health and Mobility, Vancouver, British Columbia, Canada
[5] Health, Brain, & Cognition Lab, University of Iowa, Iowa City, Iowa, U.S.A
[6] Department of Psychology, University of Iowa, Iowa City, Iowa, U.S.A
[7] Department of Psychology, University of British Columbia, Vancouver, British Columbia, Canada
[8] Department of Medicine, University of British Columbia, Vancouver, British Columbia, Canada

*Correspondence:
Dr. Teresa Liu-Ambrose, PhD, PT
Faculty of Medicine, University of British Columbia
Djavad Mowafaghian Centre for Brain Health
c/o Liu-Ambrose Lab
2215 Wesbrook Mall
Vancouver, BC V6T 1Z3
Email: teresa.ambrose@ubc.ca

Supplementary Figures
Independent Component Maps
Supplementary Figure 1. Resting-State Component Maps of Interest

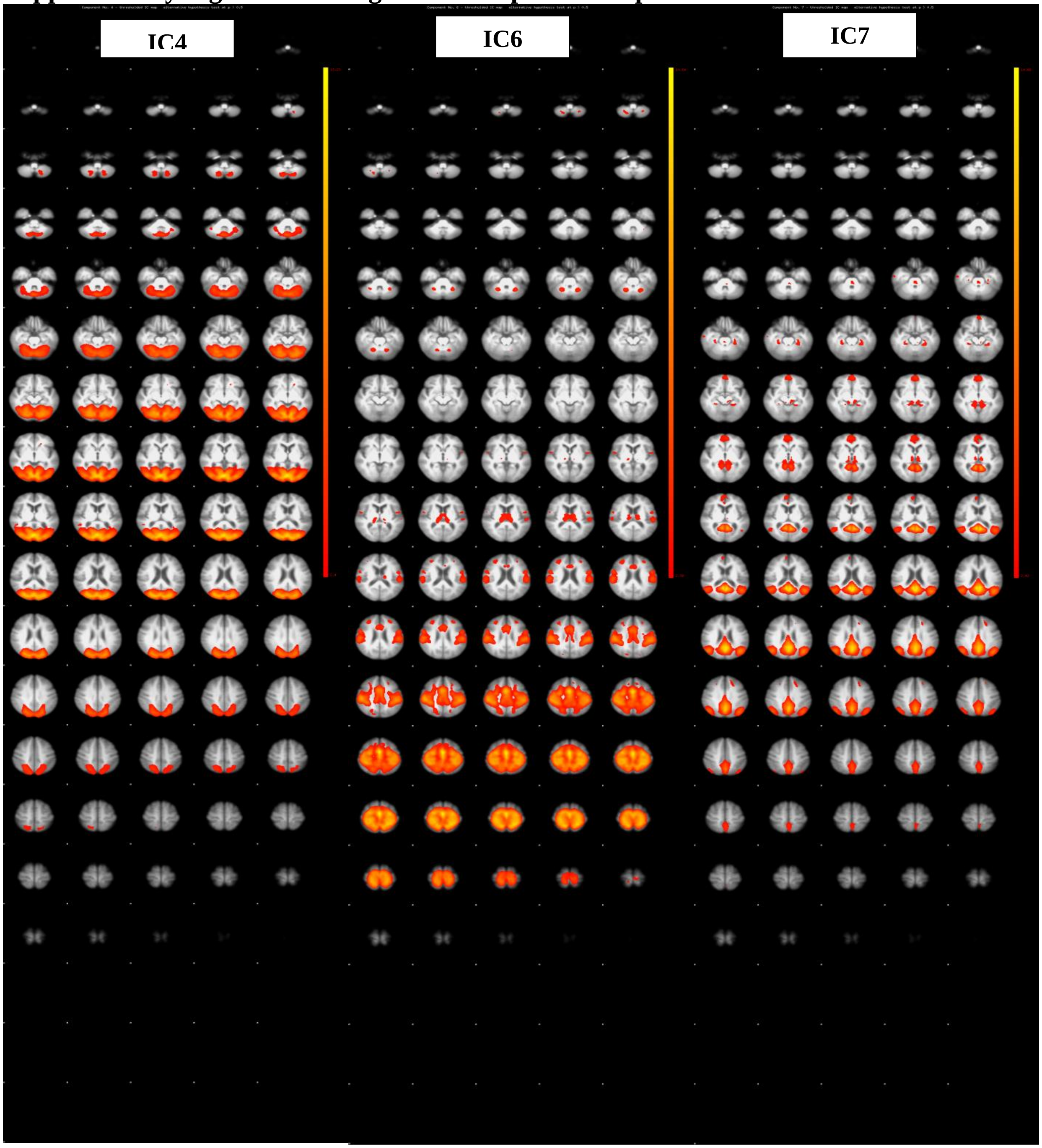

From left to right, independent component maps represent: Visual Network, Sensori-Motor Network, Default Mode Network

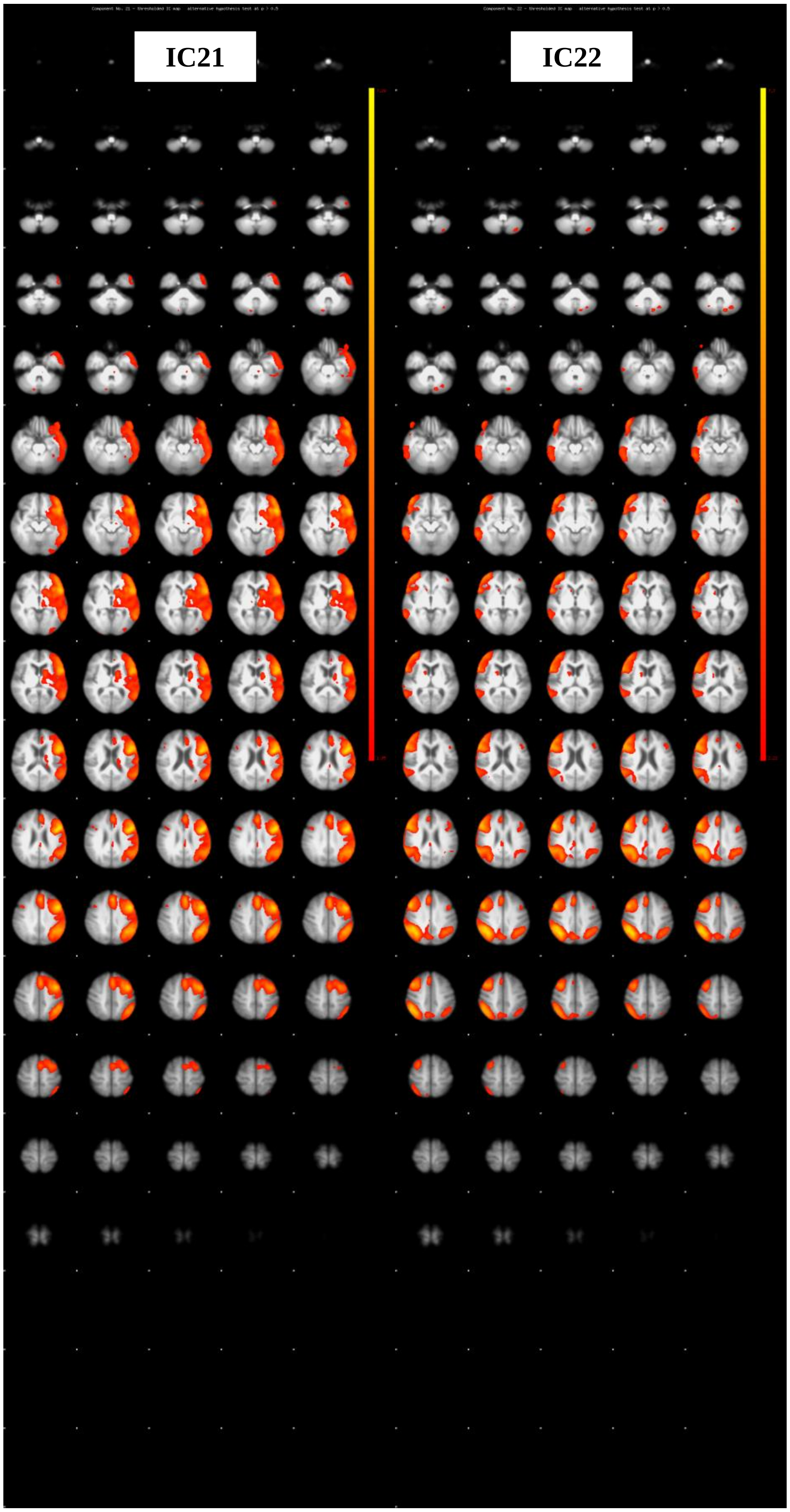

From left to right, independent component maps represent: Right Fronto-Executive Network, Left Fronto-Executive Network

Supplementary Figure 2. Task-State Component Maps of Interest

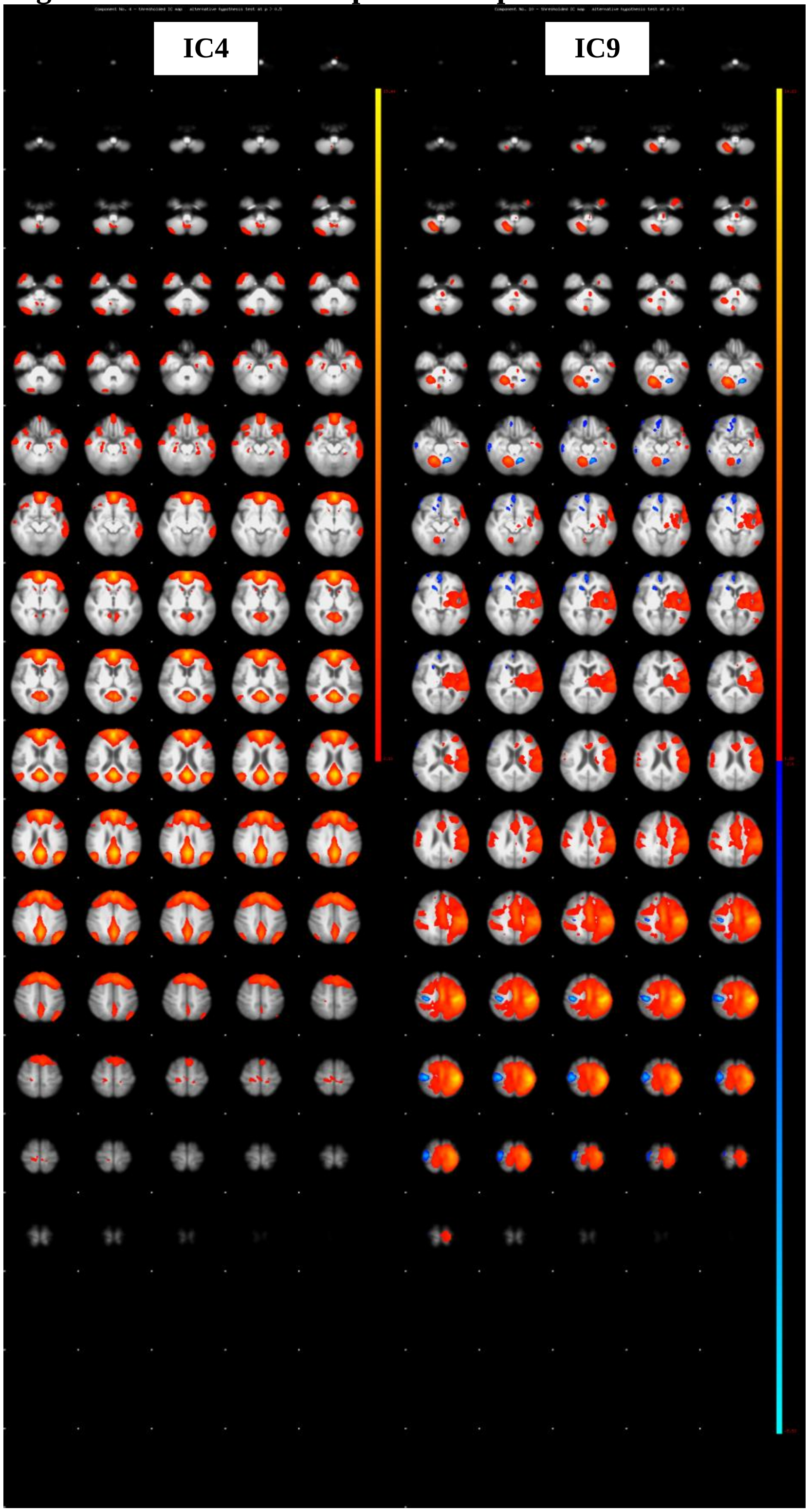

From left to right, independent component maps represent: Default Mode Network, Sensori-Motor Network

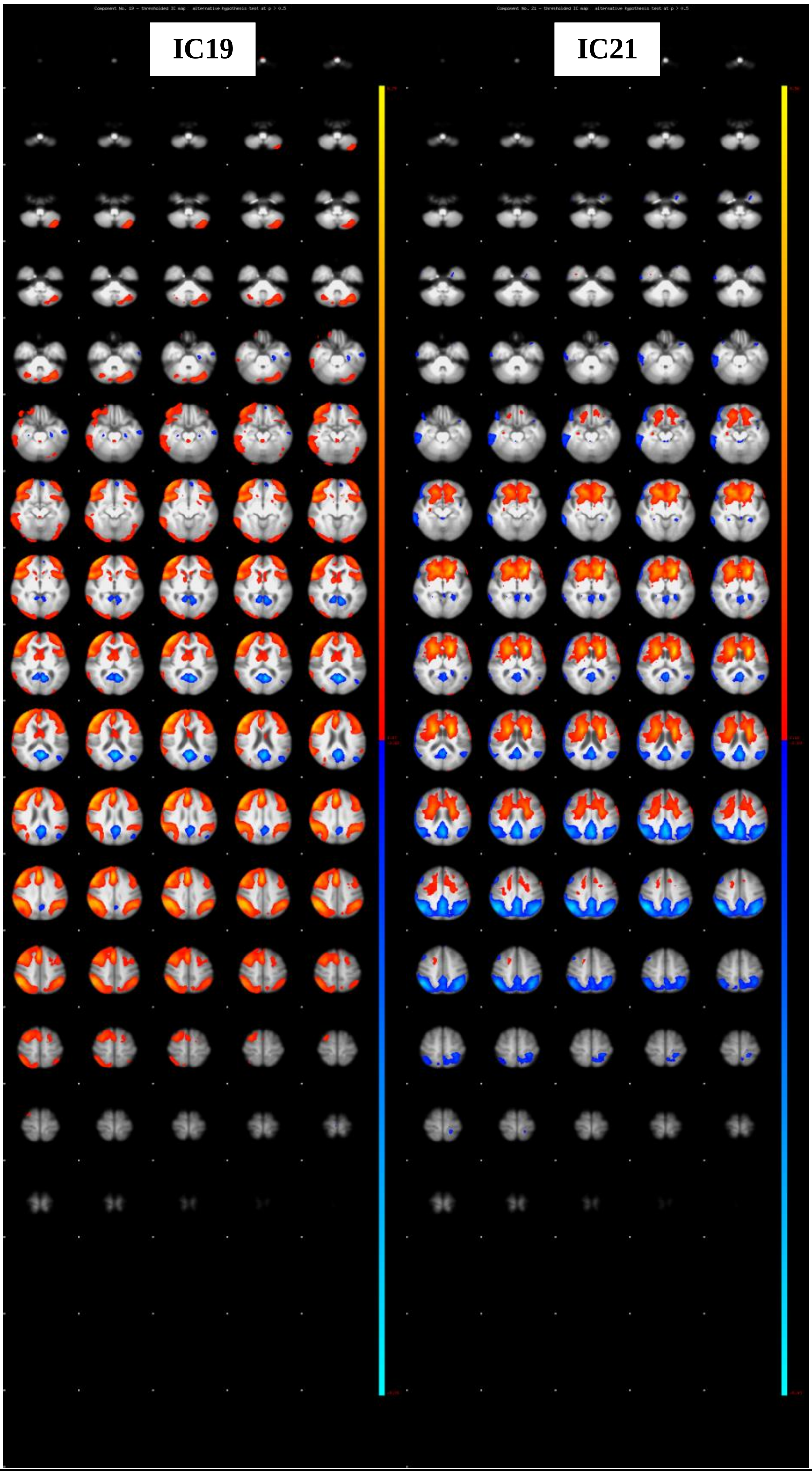

Independent component maps represent: Fronto-Executive Network,
